# Implicit Subgrid-Scale Modeling of a Mach 2.5 Spatially Developing Turbulent Boundary Layer

**DOI:** 10.3390/e24040555

**Published:** 2022-04-15

**Authors:** Guillermo Araya, Christian Lagares

**Affiliations:** High Performance Computing and Visualization Laboratory, Department of Mechanical Engineering, University of Puerto Rico, Mayaguez 00681, Puerto Rico; christian.lagares@upr.edu

**Keywords:** iLES, SUPG, supersonic, turbulent inflow conditions, boundary layers

## Abstract

We employ numerically implicit subgrid-scale modeling provided by the well-known streamlined upwind/Petrov–Galerkin stabilization for the finite element discretization of advection–diffusion problems in a Large Eddy Simulation (LES) approach. Whereas its original purpose was to provide sufficient algorithmic dissipation for a stable and convergent numerical method, more recently, it has been utilized as a subgrid-scale (SGS) model to account for the effect of small scales, unresolvable by the discretization. The freestream Mach number is 2.5, and direct comparison with a DNS database from our research group, as well as with experiments from the literature of adiabatic supersonic spatially turbulent boundary layers, is performed. Turbulent inflow conditions are generated via our dynamic rescaling–recycling approach, recently extended to high-speed flows. Focus is given to the assessment of the resolved Reynolds stresses. In addition, flow visualization is performed to obtain a much better insight into the physics of the flow. A weak compressibility effect is observed on thermal turbulent structures based on two-point correlations (IC vs. supersonic). The Reynolds analogy ($u^{\prime }$ vs. $t^{\prime }$) approximately holds for the supersonic regime, but to a lesser extent than previously observed in incompressible (IC) turbulent boundary layers, where temperature was assumed as a passive scalar. A much longer power law behavior of the mean streamwise velocity is computed in the outer region when compared to the log law at Mach 2.5. Implicit LES has shown very good performance in Mach 2.5 adiabatic flat plates in terms of the mean flow (i.e., $C_{f}$ and $U_{VD}^{+}$). iLES significantly overpredicts the peak values of u′, and consequently Reynolds shear stress peaks, in the buffer layer. However, excellent agreement between the turbulence intensities and Reynolds shear stresses is accomplished in the outer region by the present iLES with respect to the external DNS database at similar Reynolds numbers.

## 1. Introductory Remarks

The modeling of turbulent high-speed flow is crucial in supersonic/hypersonic vehicles. Among the currently available Computational Fluid Dynamics (CFD) numerical tools for tackling turbulence, the most commonly known (and cheaper) are RANS (Reynolds-Averaged Navier–Stokes), LES (Large Eddy Simulation), and DNS (Direct Numerical Simulation) [1]. DNS directly resolves the governing formulation for fluid flows called the Navier–Stokes equations; it does not employ any turbulent model but requires significant computational resources. Consequently, if one wants to carry out DNS, it is mandatory to use a highly scalable and efficient flow solver, mainly if the goal is to predict spatially developing turbulent boundary layers (SDTBL), implying accurate turbulent time-dependent inflow conditions [2,3]. On the contrary, LES models the effects of Kolmogorov scales via a spatial filter [4], and the large-scale motions are computed directly, whereas only the small-scale motions are modeled, resulting in a reduction in computational resources compared to the DNS approach. However, this computational reduction significantly depends on the analyzed geometry and flow complexity: it is well known that in wall-bounded flows, even the “inertial subrange scales” located in the near-wall region could be tiny, not to mention if detached turbulent boundary layers are going to be predicted. Thus, the computing and running effort reduction by considering LES with respect to DNS could be limited to one order of magnitude in the spatial framework, according to the present authors’ experience. This is in agreement with a more theoretically founded estimate when accounting for computational scaling in space of DNS as $O\left(Re^{9/4}\right)$ and that of LES as $O\left(Re^{13/7}\right)$ [5], which leads to roughly an order of magnitude difference at moderately high Re in space. Nonetheless, the computational cost reduction in space grows more proportional to Re. Of course, the timestep could be larger in LES for unsteady simulations and CFL constraints, introducing a new source of effort cutback. Despite its poor performance in reproducing peak values of flow fluctuations and Reynolds stresses in the buffer layer (i.e., for $y^{+}\approx$ 15–20, where turbulence production is maximum), it is a promising alternative to compute the 3D time-dependent details of the largest turbulent structures, which are responsible for most of the transport phenomena in turbulent boundary layer problems, by using a simple model for the smaller turbulent scales.

The following generic classes can be specified for LES modeling [6,7]:

- Explicit sub-grid scale (SGS) models: they assume that the numerical method provides an accurate solution to the resolved-scale equation, such as eddy-viscosity, scale-similarity, and mixed approaches. Since it operates on the smallest represented scales, it requires the numerical truncation error to be small, which could be reduced by spatial filtering.
- Implicit LES (iLES): defined as an “implicit SGS” model that is directly contained (embedded) within the numerical discretization scheme. This is very convenient and helpful for physically complex flows or complex geometries. It merges the numerical discretization with the SGS model.
  The significant difference in approach used in iLES compared with standard LES is how SGS modeling is pursued [4]. In conventional LES, a “physical” SGS model is added to the fluid dynamics calculation (or apparent stresses) to account for the unresolved scales of turbulence. The SGS model is developed based on understanding the structure and characteristics of turbulent flows. On the contrary, iLES relies upon the features of a numerical method, which abides by a set of physical principles to accomplish the same purpose as explicit LES [4]. Empirical experience has shown that iLES can achieve high-quality simulations of turbulence [8,9,10].

Furthermore, the approach of implicit LES (henceforth, iLES) has obtained significant attention on very challenging high Reynolds number flows [4] in recent years, where the use of HPC tools is almost mandatory. For instance, MILES (the Monotone Integrated LES approach) was first proposed by Boris [11] and incorporated the effects of the SGS physics on the resolved scales through functional reconstruction of the convective fluxes using locally monotonic finite volume schemes [12,13]. Thus, the MILES approach implicitly models the subgrid-scale stresses and turbulent heat fluxes through the numerical algorithm, including second-order or third-order accurate spatial schemes. Supersonic spatially developing turbulent boundary layers (SDTBL) have been predicted via explicit and implicit LES [14,15], where the rescaling–recycling technique by Lund et al. [16] was employed for turbulent inflow generation. In [14], comparisons of the MILES and Smagorinsky models exhibited almost identical results, indicating that the Smagorinsky model was not needed for supersonic turbulent boundary layers.

While, in the majority of the previously cited literature, the selected numerical techniques were finite difference (FD) or finite volume (FV), a steadily growing number of studies on turbulent boundary layers can be found via continuous finite element methods [17,18,19,20,21]. Additionally, the Discontinuous Galerkin (DG) methods (originally introduced by Reed and Hill [22] in 1973) have substantially evolved in the last few decades, gaining important ground in computational fluid dynamics [23,24]. As stated by [23], and contrary to finite volume methods, discontinuous Galerkin methods allow the use of higher-order accuracy on unstructured meshes [25,26], permitting efficient parallelization through hybridization [27,28] as well. On the other hand, DG methods exhibit superior stability and robustness concerning continuous finite element methods [23,24]. In addition, the variational multiscale (VMS) method provides a finite element-specific, mathematically rigorous approach to LES [23,29,30,31]. The availability of Sobolev subspaces allows the spatial filtering operation in LES to be defined as a projection operation [23]. More recently, Stoter et al. [23] presented a new approach for integrating discontinuous Galerkin methods in the variational multiscale paradigm for fluid dynamics problems.

In terms of iLES applied to compressible evolving wall-bounded flows, Urbin and Knight [14] successfully tested the MILES approach on an unstructured grid of tetrahedral cells over a Mach 3 adiabatic flat plate. Their numerical results proved that the subgrid-scale effects can be properly captured by MILES without needing a physical model. The subgrid-scale influence was implicitly modeled by the adaptive local deconvolution method (ALDM) in [32] to describe a supersonic turbulent boundary layer over a compression–expansion ramp configuration. Poggie et al. [33] carried out comprehensive mesh resolution and domain dimension sensitivity studies in compressible SDTBL flows via DNS and well-resolved LES, where the filtering effect of the numerical scheme supplied the needed dissipation of the unresolved scales. Flow statistics were collected over 50 non-dimensional time units ($\delta_{o}/U_{\infty }$), and the reference boundary layer thickness in wall units, $\delta^{+}$, ranged within 550–570 in all cases. Ritos et al. [34] scrutinized several high-order methods in the iLES approach for a Mach 2.25 adiabatic flat plate and $\delta^{+}$≈ 400: the Monotone Upstream-centered Schemes for Conservation Laws (MUSCL) and the Weighted-Essentially Non-Oscillatory (WENO) schemes in conjunction with the Harten-Lax-van Leer-Contact (HLCC) solver. The authors gathered 2400 samples over three flow cycles or flow-through time, obtaining excellent results in comparison with the DNS database for the ninth-order scheme; however, significant discrepancies were found in the first- and second-moment flow statistics in second- and fifth-order numerical schemes. Moreover, [35] tested three different local subgrid-scale models, including implicit LES, in adiabatic flat plates at Mach 2. The von Karman number, $\delta^{+}$, approximately ranged from 450 to 1250, and flow statistics were taken from 300$\delta_{in}/U_{\infty }$ non-dimensional time. They found a consistent near-wall trend of flow fluctuations via iLES.

In summary, in this manuscript, we evaluate for the first time the numerical performance of implicit subgrid-scale modeling provided by the well-known streamline upwind/Petrov–Galerkin stabilization (SUPG) [17,18] for the finite element discretization of advection–diffusion problems combined with the Dynamic Multiscale Approach (DMA) [36,37,38] for turbulent inflow generation in supersonic spatially developing turbulent boundary layers at a freestream Mach number, $M_{\infty }$, of 2.5 and moderate Reynolds numbers (i.e., 410 < $\delta^{+}$ < 680). Furthermore, in order to ensure a statistically steady flow, iLES was run for approximately 64 flow-through time, collecting 4001 flow fields over 1530 non-dimensional time units $\delta_{inl}/U_{\infty }$, which is a significantly larger sampling time as compared to previous iLES work.

## 2. Governing Equations and Inflow/Boundary Conditions

In this work, the flow can be safely assumed to follow a continuum formulation given the very low Knudsen number. Furthermore, the conditions present allow for the assumption of local equilibrium due to the lack of chemical reactions, strong shocks, and other high enthalpy effects. By assuming local equilibrium and a continuum, we also neglect molecular vibration and rotation. After these assumptions, we arrive at the classical compressible Navier–Stokes equations [39], which are a set of non-linear partial differential equations. Given the compressible nature of the flow, we can no longer assume trivial conservation of mass by equaling the flow divergence to zero because abrupt density variations are seen due to pressure waves. To this end, we apply the weak form of the equations presented in Equations (1)–(3) (although presented in their strong conservation form for clarity and brevity).

(1) $$\frac{\partial \rho }{\partial t}+\frac{\partial }{\partial x_{j}}\left(\rho u_{j}\right)=0$$

(2) $$\frac{\partial \rho u_{i}}{\partial t}+\frac{\partial }{\partial x_{j}}\left(\rho u_{i}u_{j}+p\delta_{ij}-\sigma_{ij}\right)=0$$

(3) $$\frac{\partial \rho e}{\partial t}+\frac{\partial }{\partial x_{j}}\left(\left(\rho e\right)u_{j}-u_{i}\sigma_{ij}+q_{j}\right)=0$$

The equations follow index notation, where *i* refers to the *i*-th coordinate. Further, ρ is the density calculated from the ideal gas law (under the assumption of a calorically perfect gas); *p* is the pressure; $\sigma_{ij}$ is the viscous stress tensor. In this work, we assume that the stress tensor follows that of a Newtonian fluid (i.e., a linear stress–strain law).

(4) $$\sigma_{ij}=\mu \left(\frac{\partial u_{i}}{\partial x_{j}}+\frac{\partial u_{j}}{\partial x_{i}}\right)-\frac{2}{3}\mu \delta_{ij}\left(\frac{\partial u_{k}}{\partial x_{k}}\right)$$

In Equation (4), μ is defined as the dynamic viscosity, and $q_{i}$ encompasses the heat fluxes due to thermal gradients in the *i* direction. In this work, we assume that heat fluxes follow Fourier’s law, $q_{i}=\kappa \frac{\partial T}{\partial x_{i}}$, where κ is the thermal conductivity. Finally, *e* in Equation (3) is the total energy per unit mass, which we assume to follow:

(5) $$e=c_{v}T+\frac{1}{2}u_{i}u_{j}$$

As presented in Equation (5), $c_{v}$ is the specific heat at constant volume. Finally, we model the fluid viscosity following a power law as per Equation (6).

(6) $$\mu =\mu_{\infty }{\left(\frac{T}{T_{\infty }}\right)}^{0.76}$$

As we previously hinted, the finite element flow solver used for the present work implements the equations in their weak form. The PHASTA flow solver [40] was chosen due to its computational efficiency, strong scaling performance, and validated implementation of the governing equations. The Petrov–Galerkin (SUPG) finite element spatial discretization scheme is the basis for the scheme used in this work. The SUPG scheme implementation offers second-order accuracy in space [17,18]. Moreover, we employ a fully implicit, second-order discretization in time. Numerical dissipation is employed as an implicit subgrid-scale (SGS) model. A high-performance, iterative Krylov solver is used to solve the resulting system of equations in space. We will not delve further into the details regarding the finite element method employed; however, interested readers are referred to [41,42].

The SDTBL’s physics modeling via LES must take into account the following key points:

1. The computational domain ought to be sufficiently large to contain even the so-called “superstructures” or largest scale motions (LSM) (Hutchins and Marusic [43,44]),
2. Injecting accurate, time-dependent inflow turbulent fluctuations is required to reduce the streamwise computational domain length since the flow is no longer required to develop from freestream conditions. This allows for the simulation of larger-scale systems (i.e., larger Re) [37,45].
3. It is indispensable that the turbulent inflow information exhibits a physically accurate power spectrum to minimize the “inlet developing section” and reduce the “non-physical” developing section [46] (ideally, this section should be as small as possible, but can be typically reduced to the order of 2–3 $\delta_{inl}$’s).

**Figure 1:**
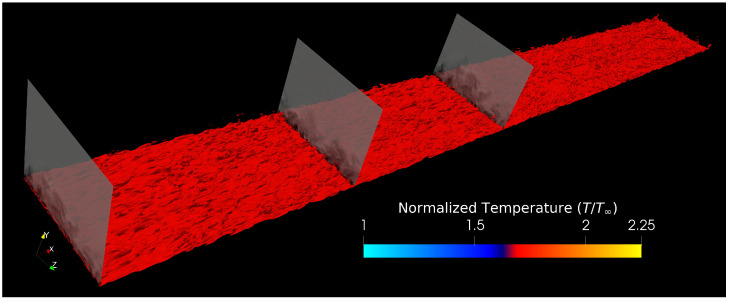
Boundary layer schematic for the supersonic turbulent boundary layer via iLES. Iso-surface of normalized instantaneous static temperature ($T/T_{\infty }=$ 1.7). Contours of velocity magnitude in cross-sectional planes (i.e., inlet, test, and recycle plane).

To address aforementioned points 2 and 3, we use a modified version of the inflow condition generation approach proposed by Araya et al. [45], the Dynamic Multiscale Approach (DMA), extended to account for compressibility effects in SDTBLs in [36,37,38]. The DMA is based on a modified version of the approach proposed by Lund et al. [16], widely known as the rescaling–recycling approach. Furthermore, it has already been demonstrated that the compressible DMA can dramatically shorten the development section (see Lagares and Araya [46]) to, at most, ∼$2.5\delta_{inlet}$. Moreover, it has been shown that it is capable of reproducing inflow conditions, preserving the qualities of a developed flow’s energy spectra [46]. Interestingly, the same work showed that the outflow condition could have a more drastic effect on the overall quality of the power spectra [46]. Xu and Martin [47], Urbin and Knight [14], and Stolz and Adams [15] have also proposed extensions to Lund’s original methodology for compressible boundary layers. Contrary to previous approaches, and as will be addressed later in the manuscript, we avoid the need for empirical correlations in connecting the recycle plane’s friction velocity to the inlet’s friction velocity. To aid in the discussion of the DMA, we provide an infographic depicting iso-contours of the outer-scaled instantaneous static temperature on the method in Figure 1. The idea behind the rescaling–recycling method can be succinctly stated as follows: “re-insert a scaled flow solution extracted from a recycled plane downstream”. Others have reported that prescribing a steady, Dirichlet pressure inlet condition results in higher stability and accuracy in numerical cases than an unsteady, fluctuating pressure. We have also found that a steady pressure at the inlet yields a more accurate and stable numerical simulation. Both Kistler and Chen [48] and Urbin and Knight [14] state that “the static pressure can be assumed constant at the inlet plane since the pressure fluctuations are small compared to the static temperature fluctuations”. Note that we indirectly impose instantaneous density profiles due to the calorically perfect gas assumption, leading to the perfect gas equation of state. The core idea behind the transformations (i.e., scaling laws) is to transform the streamwise, non-homogeneous flow conditions into quasi-homogeneous conditions. We follow a statistical description of the instantaneous flow parameters based on the Reynolds decomposition:

(7) $$u_{i}\left(x,t\right)=u_{i}^{\prime }\left(x,t\right)+{\bar{U}}_{i}\left(x,y\right)$$

(8) $$T\left(x,t\right)=t^{\prime }\left(x,t\right)+\bar{T}\left(x,y\right)$$

As part of the inflow condition generation procedure, we must consider the SDTBL as two zones, an inner and an outer portion, that blend smoothly. To this end, we apply distinct scaling laws for these two zones [45], hence the term multiscale in the Dynamic Multiscale Approach. We blend the two via a smooth, blending function, thus creating a composite, instantaneous flow profile accounting for phenomena in both zones. The projection preserves dimensionless wall distances by mapping values at iso-$y^{+}$ in the inner zone and at iso-y/δ in the outer zone. As was previously mentioned, Figure 1 shows thermal iso-contours in the computational domain while also highlighting the inlet, test and recycle planes. When re-scaling the flow parameters [45], we must account for the ratio of the friction velocity, $u_{\tau }$, at the inlet to that of the recycle plane. We define $u_{\tau }$ as $\sqrt{\tau_{w}/\rho }$, where ρ is the fluid density and $\tau_{w}$ is the shear stress at the wall. Given that we must prescribe the inlet boundary layer thickness per the predicted Reynolds number at the inlet, prescribing $u_{\tau ,inl}$ is not only unnecessary but redundant. Seeking to tackle this issue, Stolz and Adams [15], Urbin and Knight [14], and Lund et al. [16] have used the 1/8-th power law relating the momentum thickness in ZPG flows to the friction velocity as: $u_{\tau ,inl}/u_{\tau ,rec}={\left(\delta_{2,inl}/\delta_{2,rec}\right)}^{-1/8}$. This empirical power law can strongly depend on the Reynolds number and other compressibility effects. To this end, [45] introduced a dynamic component (hence the “dynamic” in DMA) calculating the power law’s exponent dynamically by connecting a new, “test” plane (mentioned in Figure 1) to the recycle plane as:

(9) $$\gamma_{\delta 2}=\frac{ln(u_{\tau ,test}/u_{\tau ,rec})}{ln(\delta_{2,test}/\delta_{2,rec})}.$$

In Figure 1, one can observe typical “bulges” and “valleys” as in incompressible thermal turbulent boundary layers, with the outer irrotational flow penetrating further into the near-wall region.

The rest of the boundary conditions in computational domains are as follows: the standard no-slip condition is imposed for all velocity components at the bottom surface. In supersonic cases at Mach 2.5, quasi-adiabatic conditions were prescribed at the wall. The ratio $T_{w}/T_{\infty }$ is 2.25 and $T_{w}/T_{r}$ is 1.06, where $T_{w}$ is the wall temperature, $T_{\infty }$ is the freestream temperature, and $T_{r}$ is the recovery or adiabatic temperature. The Prandtl number (Pr) was set to 0.72 in the compressible cases. The temperature is considered a passive scalar (Pr = 0.71) for the incompressible case plus isothermal wall conditions. Periodicity of all instantaneous flow variables is imposed on lateral surfaces. On the top surface, freestream values are imposed. At the outflow plane, nothing is prescribed, but flow parameters are extrapolated from the interior of the physical domain.

### 2.1. Computational Domain and Discretization Strategies

**Figure 2:**
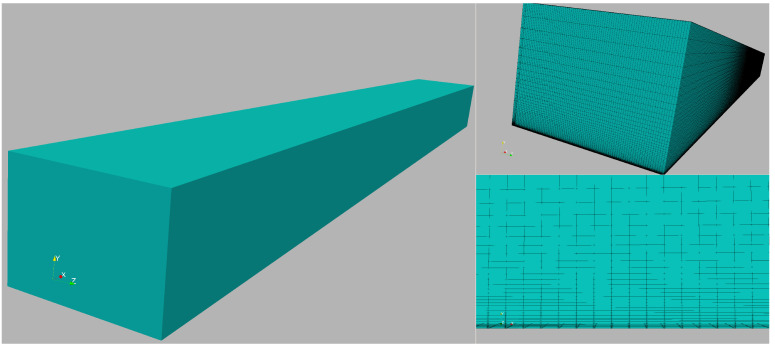
A 3D isometric view of the computational box (**left**), inflow plane with hexahedral element distribution (**right**, **top view**), and close-up of the near-wall discretization (**right**, **bottom view**) in the iLES case.

In this section, details of the computational domain, discretization strategies, and influence of the selected mesh on numerical results are shown and discussed for the iLES case. In Figure 2 (left image), an isometric view of the computational box is seen. In addition, the streamwise dimension of the computational box is chosen to be large enough (in the order of forty boundary layer thicknesses) to properly capture the large-scale motions (LSM) or “superstructures” that carry most of the turbulent energy of the flow and whose streamwise length scales are at least six boundary thicknesses, as discussed by [43]. Furthermore, the domain width is prescribed as approximately four and a half inlet boundary layer thicknesses. According to our previous experience and the generally accepted spanwise length, the selected domain width can gather several low/high-speed streaks, as shown and discussed later in this section. In the upper right image of Figure 2, a foreground view of the inlet plane is visualized. Hexahedral elements (2,046,179 elements in total) have been considered in the full domain, showing excellent performance in boundary layer problems and minimal numerical dissipation. The reader is referred to Table 1 for further details regarding mesh resolution and the number of grid points for this case. The lower right image of Figure 2 depicts a close-up of the hexahedral element distribution. Approximately 75% of the wall-normal points were clustered inside the turbulent boundary layer. The mesh is equidistant in the streamwise and spanwise direction but stretched in the wall-normal or vertical direction with a growth factor of roughly 1.1.

**Figure 3:**
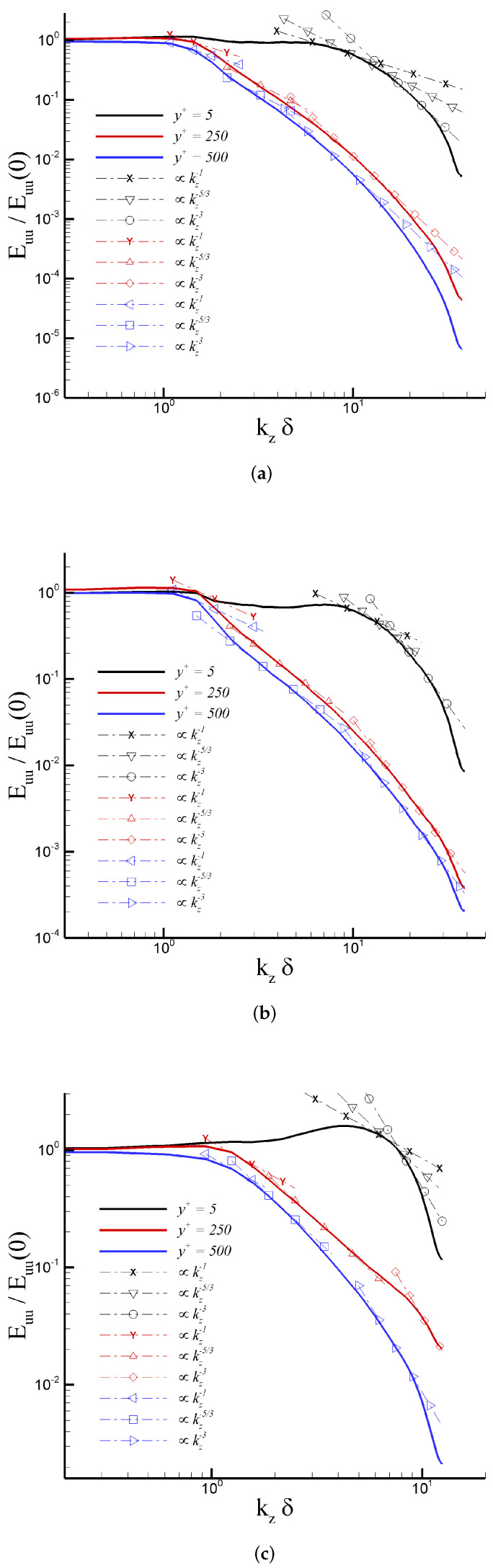
One-dimensional energy spectra in the spanwise direction at $y^{+}=$ 5, 250, and 500 for: (**a**) Incompressible DNS, (**b**) Mach 2.5 DNS, and (**c**) Mach 2.5 iLES.

As stated by Tejada-Martinez and Jansen [21], when dealing with LES, the resulting outcomes are determined by the numerical approach and grid point distribution employed. Thus, performing “grid refinement to achieve grid-independent results would lead to a DNS, no longer LES”. Therefore, the impact of the mesh on our numerical results is evaluated by computing the one-dimensional energy spectra ($E_{uu}$) and two-point correlations ($R_{uu}$) of the streamwise velocity fluctuations ($u^{\prime }$) in the spanwise direction for the numerical cases considered in this study. Figure 3 exhibits the power spectra ($E_{uu}$/$E_{uu}\left(0\right)$) normalized by the energy at the first wavenumber at three different wall-normal stations, i.e., $y^{+}=$ 5, 250, and 500. The wall-normal coordinates were strategically selected to scrutinize mesh resolution in the linear viscous layer ($y^{+}=$ 5), in the middle of the log region ($y^{+}=$ 250), and in the wake region ($y^{+}=$ 500) of the turbulent boundary layer. DNS cases for the incompressible regime (Figure 3a) and the supersonic regime (Figure 3b) show a significant drop off of the energy spectra (up to five decades). Additionally, the energy-containing scale (∼$k_{z}^{-1}$) and the typical −5/3 inertial range (∼$k_{z}^{-5/3}$) are clearly identified. In the dissipation range at the highest wavenumbers, smooth “tails” indicate a proper capture of the Kolmogorov length scales. Furthermore, in this high-wavenumber zone, the spectra do not show any energy pile-up, confirming that turbulence scales are appropriately resolved [49]. Interestingly, we have detected the presence of a considerable zone with a local slope ∼$k_{z}^{-3}$, immediately down the −5/3 inertial range or energy transfer range. According to [50], a second inertial range (i.e., enstrophy transfer range) can be found in two-dimensional turbulence. In particular, an evident enlargement of the inertial range with slope ∼$k_{z}^{-3}$ can be observed as one moves further from the wall. However, further analysis must be performed to physically explain this spectrum’s behavior in three-dimensional turbulence, which is beyond the scope of the present manuscript and will be published elsewhere. Turning to iLES energy spectra based on resolved streamwise velocity fluctuations, while the total drop-off of energy spectra is smaller than that of the DNS cascade, the major aspects and features are present. Moreover, the inertial sub-range has been acutely reproduced, implying a suitable grid point distribution in the iLES domain. The mesh resolution was prescribed based on typical values [15].

**Figure 4:**
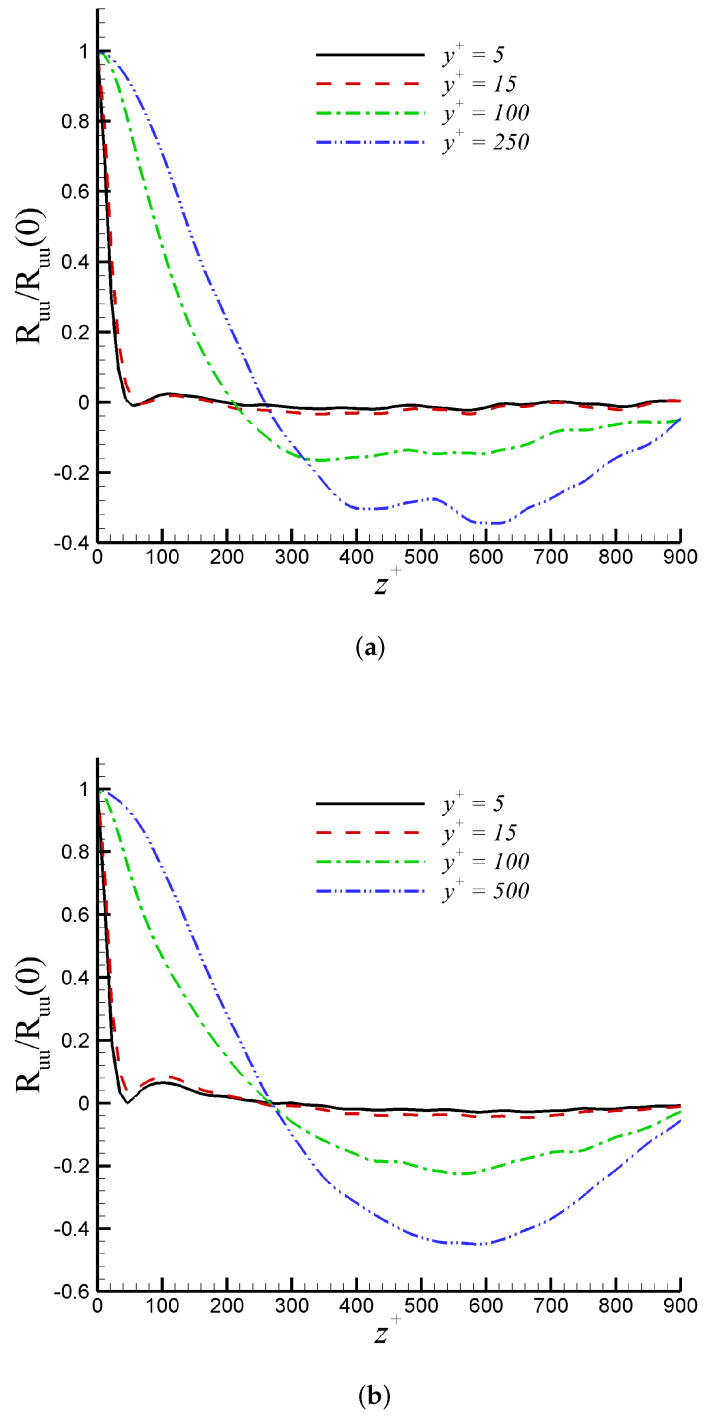

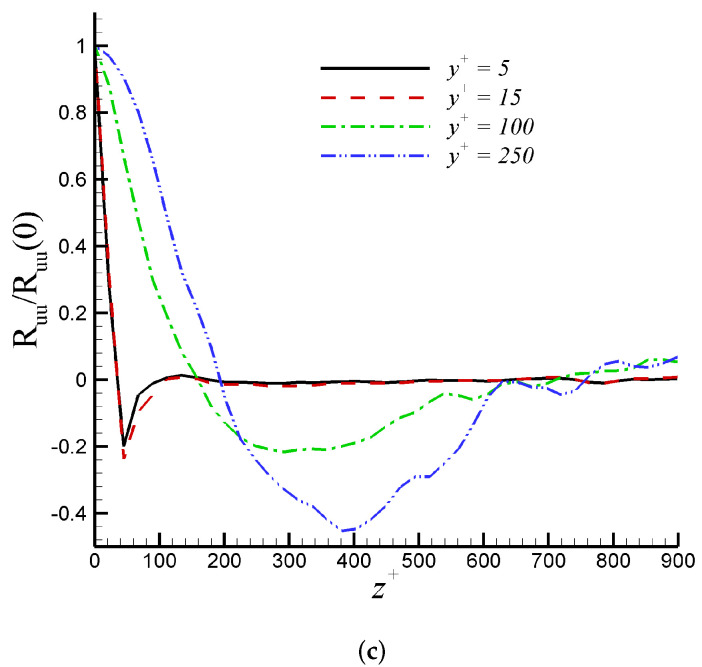
Two-point correlations $R_{uu}$ in the spanwise direction at $y^{+}=$ 5, 15, 100, and 250 for: (**a**) Incompressible DNS, (**b**) Mach 2.5 DNS, and (**c**) Mach 2.5 iLES.

Davidson [51] suggested in his paper that two-point correlations were the best statistical parameter for assessing LES resolution. The two-point correlations ($R_{uu}$) of $u^{\prime }$ are shown at $y^{+}=$ 5, 15, 100, and 250 in Figure 4 for DNS and iLES. In all cases, it is observed that the autocorrelation coefficients, $R_{uu}$, decay toward zero over a distance of $L_{z}/2$ at most in the outer region. This confirms that the computational domain is sufficiently wide along the spanwise direction. Additionally, the profiles of $R_{uu}$ in Figure 4 depict a local minimum. For $y^{+}=$ 5 and 15, these minimum values are at $z^{+}\approx$ 50, which indicates an average spacing ($\lambda^{+}=\lambda u_{\tau }/\nu$) of low-speed streaks equal to 100. This value is in agreement with the universally accepted range of $\lambda^{+}=100\pm 20$ according to [52]. An evident streak spacing increase can be seen in the log region. The trend of two-point correlations confirms the suitability of the selected domain width in all cases. This is consistent with the resolution sensitivity study by [33], stating that the computational domain should be at least two times the maximum boundary layer thickness in iLES predictions. In our case, the iLES computational domain is 2.7$\delta_{max}$.

**Table 1:** Numerical cases.

Case	Approach	$M_{\infty }$	$T_{w}/T_{\infty }$	Reδ2	$\delta_{\max}^{+}$	$L_{x}$×$L_{y}$×$L_{z}$	$\Delta x^{+}$,$\Delta y_{\min}^{+}$/$\Delta y_{\max}^{+}$, $\Delta z^{+}$	Cores	$N_{x}$×$N_{y}$×$N_{z}$
Incompressible (IC)	DNS	0	Isothermal	2000–2400	951	$16\delta_{inl}\times 3\delta_{inl}\times 3\delta_{inl}$	11.5, 0.4/10, 10	1200	990 × 250 × 210
Supersonic Q-adiabatic	DNS	2.5	2.25	2867–3406	966	$15.1\delta_{inl}\times 3\delta_{inl}\times 3\delta_{inl}$	11.9, 0.4/11, 12	1200	990 × 250 × 210
Supersonic Q-adiabatic	iLES	2.5	2.25	1310–2141	680	$43\delta_{inl}$× 3.3$\delta_{inl}$ × 4.4$\delta_{inl}$	40.4, 0.53/49, 22.4	96	440 × 60 × 80

### 2.2. The Flow Solver for HPC

Performing Direct Numerical Simulations or Large Eddy Simulations at high Reynolds numbers such as the ones included in the present work requires a very efficient, highly scalable CFD solver. In this work, we leveraged the open-source PHASTA flow solver. The interested reader is referred to [17,18,41] for a more in-depth overview of PHASTA and its use in both incompressible and compressible flows. Combining minimal dissipation numerics and adaptive [53,54,55] unstructured meshes, PHASTA has been applied to flows ranging from validation on DNS and LES benchmarks such as channel flow and decay of isotropic turbulence [17,20,21,56] to cases of practical interest including incompressible ([19,57] and compressible [37,55,58,59,60] boundary layer flow control and hypersonic flows [36,38]. As a result, PHASTA has a strong track record of supporting closely coordinated experimental–computational studies [55,57,58,59,60]. PHASTA leverages implicit techniques to bridge the gap across a broad range of length and time scales in many flow regimes, including turbulent flows based on multiple numerical approximations, including Unsteady RANS (URANS), Detached Eddy Simulations (DES), LES, and DNS [18]. Furthermore, PHASTA’s performance and strong scaling have been studied in depth on high-performance computers [19,61,62].

## 3. Mean Flow and Higher-Order Statistics

Table 1 shows details of the cases to be discussed in this section. A supersonic DNS database at Mach 2.5 will be used as a validation tool for supersonic iLES. In addition, an incompressible DNS database is utilized to assess compressibility effects. More information about these DNS cases at high Reynolds numbers can be found in Lagares and Araya [3]. For the incompressible case, the wall temperature condition is isothermal, while it is quasi-adiabatic for both supersonic cases with a wall temperature to freestream temperature ratio of 2.25 (=$T_{w}/T_{\infty }$). Similarly, we enforce a wall temperature to the adiabatic wall temperature (recovery temperature) ratio of 1.06 (=$T_{w}/T_{r}$). Table 1 also exhibits the compressible momentum thickness Reynolds numbers ($Re_{\delta 2}=\rho_{\infty }\delta_{2}U_{\infty }/\mu_{w}$) and the friction Reynolds number or von Karman number ($\delta^{+}$ = $\delta u_{\tau }/\nu_{w}$). Here, $\delta_{2}$ is the compressible momentum thickness, $U_{\infty }$ is the freestream fluid velocity, $\rho_{\infty }$ is the freestream fluid density, $\mu_{w}$ is the dynamic viscosity at the wall, δ is the 99% boundary layer thickness, and $u_{\tau }$ is the friction velocity. Finally, information regarding domain dimension, mesh resolution in wall units, the number of cores employed, and the number of grid points considered is depicted in Table 1, as well. The ratios of the maximum to the inlet boundary layer thickness, $\delta_{max}/\delta_{inl}$, are 1.19, 1.2, and 1.65 for the incompressible DNS, supersonic DNS, and supersonic iLES cases, respectively. Sampling collection for statistical analysis is based on averaging 4001 flow fields in all cases over 302, 295, 1530 non-dimensional time units $\delta_{inl}/U_{\infty }$ for the incompressible DNS, supersonic DNS, and supersonic iLES cases, respectively, where $\delta_{inl}$ is the inlet boundary layer thickness. Samples were taken after at least 15 flow-through times to allow the flow to evolve. We consider $\delta_{inl}$ as the length scale for time normalization purposes as well as an adequate statistical collection time, as done by [49]. In their study, [49] performed DNS of supersonic turbulent boundary layers at $M_{\infty }=$ 2 and collected statistical samples over 333 and 242.8 non-dimensional time units $\delta_{inl}/U_{\infty }$ for TBL2 and TBL3 cases at similar Reynolds numbers as considered here. The present domain widths, relative to the maximum boundary layer thickness, range over $L_{z}/\delta_{max}$ = 2.5, 2.42, and 2.45, for the incompressible DNS, supersonic DNS, and supersonic iLES cases, respectively. These values are consistent with recommendations by [33], where it was concluded that the domain width must be at least two times the maximum boundary layer thickness to sufficiently capture low/high-speed streaks as well as spectral energy content in iLES.

**Figure 5:**
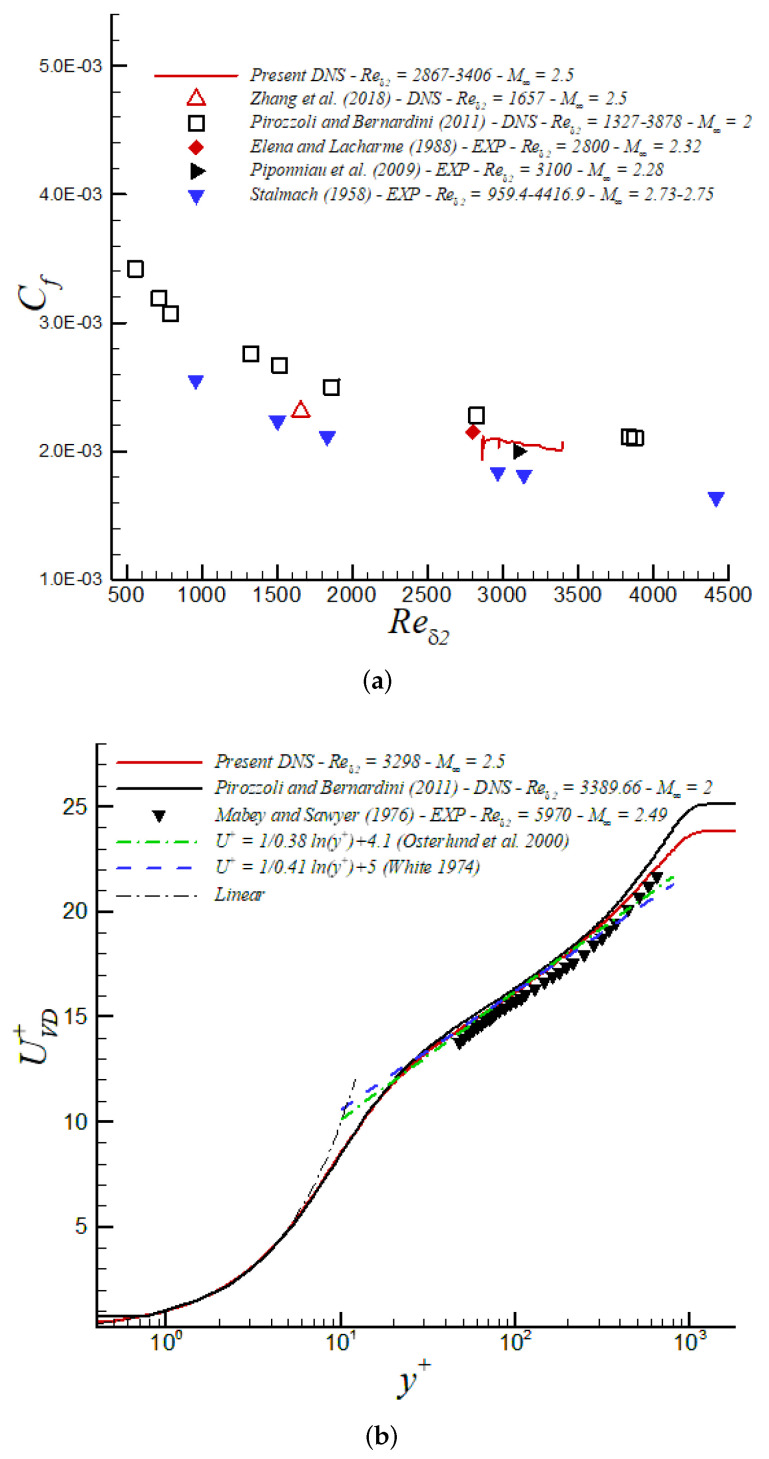
(**a**) Skin friction coefficient and (**b**) van Driest transformation in wall units at $M_{\infty }$ = 2.5 via DNS.

In Figure 5a, we highlight the skin friction coefficient including data for our aforementioned DNS data under supersonic conditions at high Re numbers [3]. Excellent agreement between the computational [3] and experimental [63,64] results is evident. Further, we can observe in Figure 5b the inner-scaled streamwise velocity profile, where the compressible results are plotted following the van Driest transformation. We observe excellent agreement between our present numerical results and experimental data from Mabey and Sawyer [65], albeit at a slightly higher $Re_{\delta 2}$ and with a minor difference in Mach number at Mach 2.49. Furthermore, the present DNS data show a high degree of collapse with DNS data by Pirozzoli and Bernardini [49] at a similar Reynolds number but lower Mach at $M_{\infty }=2$. This suggests that the van Driest transform has absorbed weak compressibility effects at these rather moderate Mach numbers. Moreover, we observe that the log-law coefficients proposed by White [66] and Osterlund et al. [67] tend to describe the large logarithmic region (≈80 wall units) for our DNS at $Re_{\delta 2}$ = 3298.

**Figure 6:**
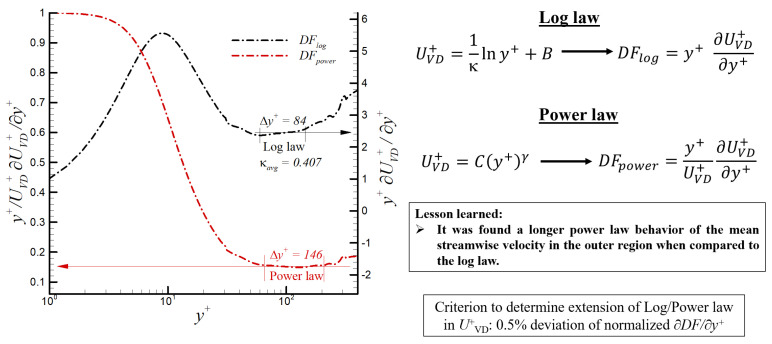
Diagnostic functions for log and power law in the supersonic regime ($M_{\infty }$ = 2.5) via DNS.

The infographic in Figure 6 depicts the performed analysis to evaluate the presence of either logarithmic or power law inside the supersonic SDTBL based on highly accurate DNS data. In Figure 6, the corresponding diagnostic functions for the log and power laws are highlighted. Constant values of these functions indicate the local presence of either a log or power law behavior. Moreover, an average value of 0.407 was obtained for the κ or von Karman constant. On the other hand, the computed average value of γ in the power law was approximately 0.15. Interestingly, the power law region occupies a more extended portion of the boundary layer with respect to the log law, i.e., 146 (power) vs. 84 (log) in wall units.

**Figure 7:**
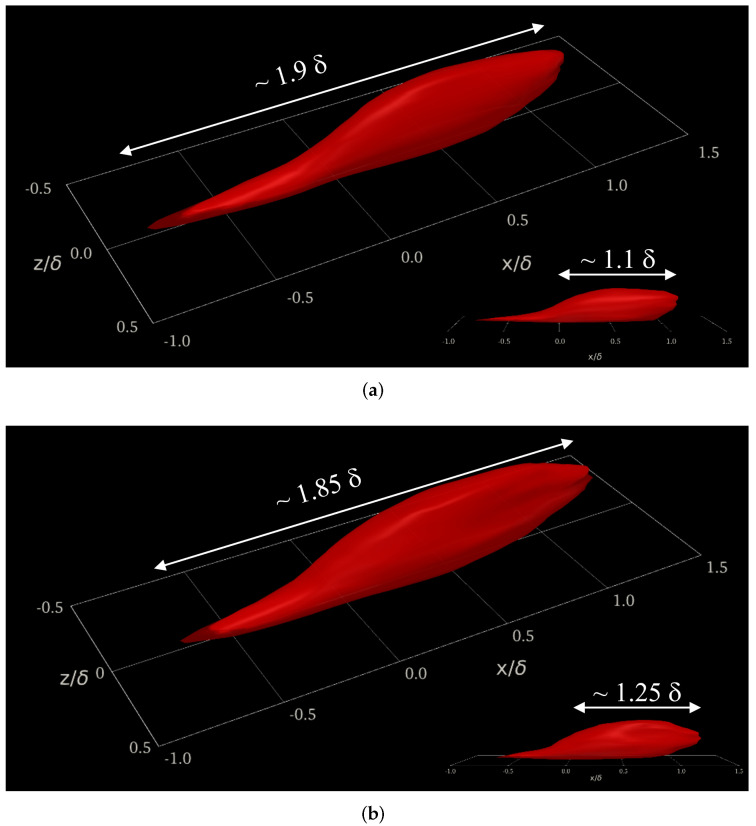
The 3D two-point correlation of thermal fluctuations ($R_{tt}$) at $y^{+}=15$. (**a**) Incompressible, (**b**) supersonic.

**Figure 8:**
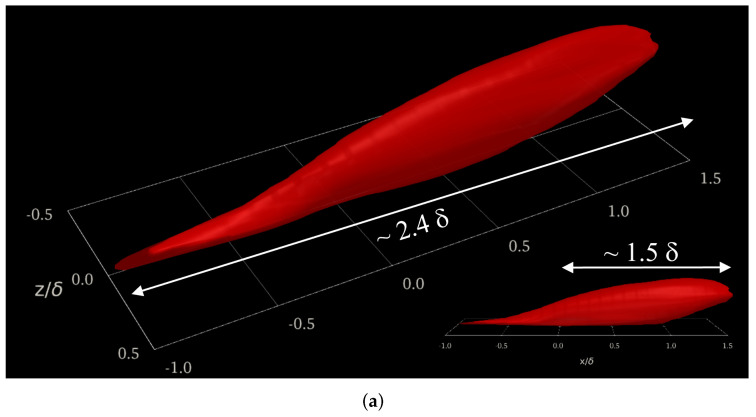

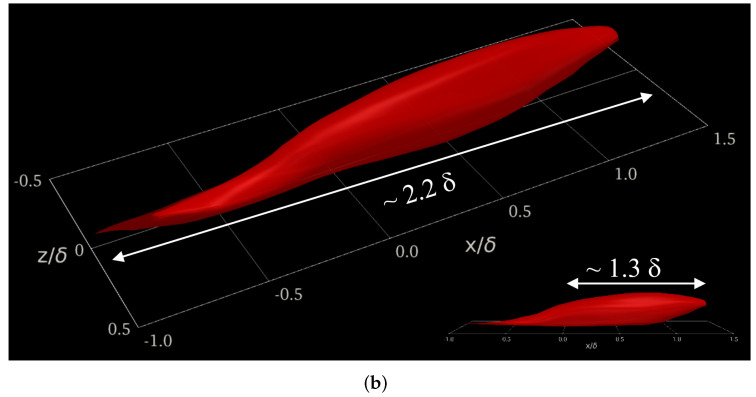
The 3D two-point cross-correlations of u′t′ ($R_{ut}$) at $y^{+}=15$. (**a**) Incompressible, (**b**) supersonic.

The scope of this work extends to the compressibility effects on velocity and temperature two-point correlations in the buffer region ($y^{+}\approx 15$). We focused on this region due to the strong turbulence and large mean velocity gradients present due to the peak turbulence production and peak values of streamwise velocity ($u^{\prime }$) and thermal ($t^{\prime }$) fluctuations. Due to the previously mentioned peak turbulence production and intensity, the buffer region is often characterized by small, highly energetic length scales in addition to low/high-speed streaks and maximum energy transfer from the mean components to the turbulent kinetic energy (i.e., the fluctuating components). The time-averaged two-point correlations of thermal fluctuations, $R_{tt}$, are contrasted in Figure 7 along the streamwise, wall-normal, and spanwise axes for DNS cases. Incompressible and supersonic thermal fluctuations in the reference zone (i.e., x/δ=0, $y^{+}=15$ and z/δ=0, being δ the local momentum boundary layer thickness) depict a strong correlation with downstream temperature fluctuations, showing long “heads” larger than 1δ. This downstream influence zone of thermal fluctuations is more preponderant in the supersonic regime, as indicated by the white arrows in the inserts of Figure 7. Notice that a slightly thicker structure is observed for the supersonic regime while showing a longer streamwise “head” but shorter “tail” than the turbulent structure of the incompressible regime. In general, both turbulent thermal structures for the incompressible and supersonic cases exhibit a similar streamwise length (around 1.85–1.9 δ) and analogous spanwise width ∼0.12–0.13δ’s (not shown). Furthermore, the supersonic effects on the Reynolds analogy (i.e., by analyzing the correlation between streamwise velocity fluctuations, $u^{\prime }$, and thermal fluctuations, $t^{\prime }$) are scrutinized by computing time-averaged two-point cross-correlations $R_{ut}$, as seen in Figure 8. Both parameters depict a high level of correlation, with total streamwise lengths in the order of 2.2–2.4δ’s. The similarity between iso-surfaces of $R_{ut}$ in the incompressible and supersonic regime is impressive (let us clarify that $R_{ut}$ < 0 in the supersonic SDTBL, which has been normalized by the reference value $R_{ut,o}$), indicating that the Reynolds analogy possesses a weak effect of the Mach number (compressibility), at least for the conditions (Reynolds number and wall adiabatic conditions) considered in our DNS approach [3]. Let us recall that the temperature in the incompressible case was assumed to be a passive scalar with isothermal wall conditions.

**Figure 9:**
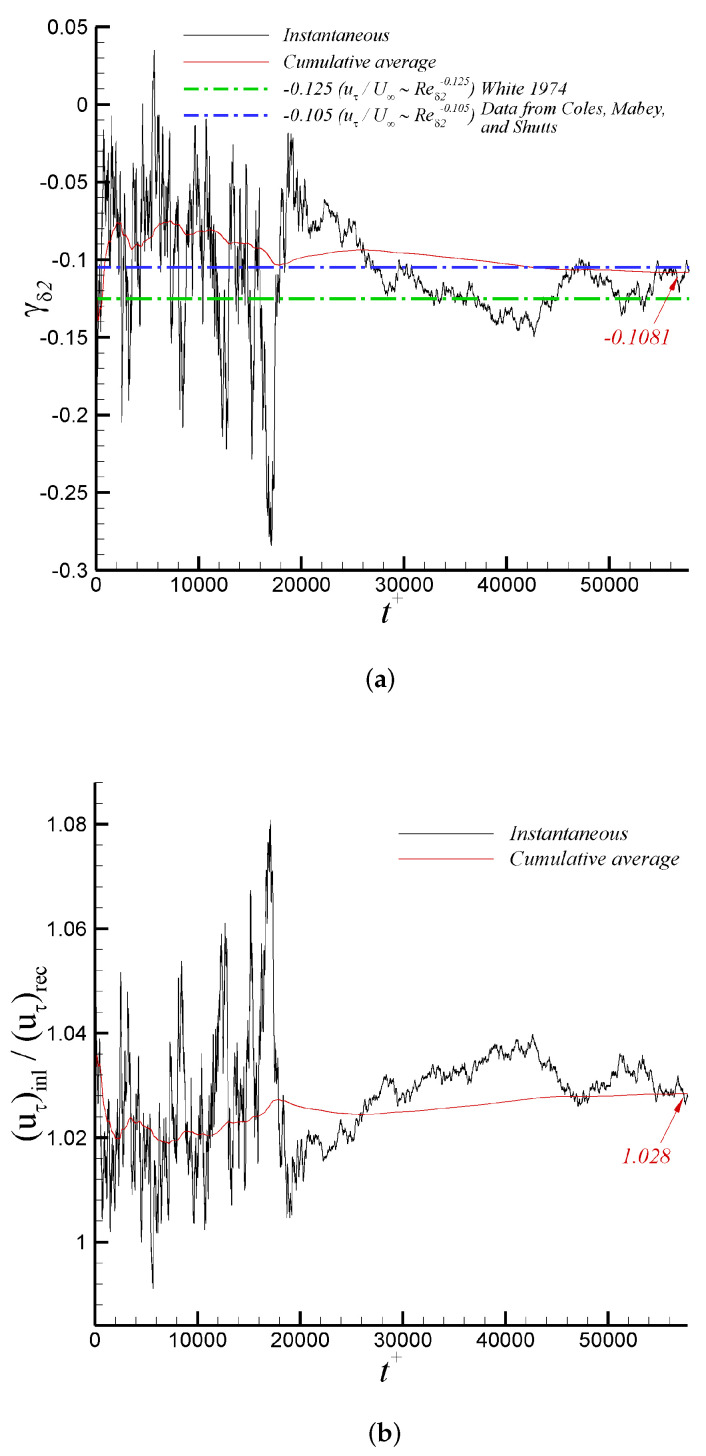
Time variation of (**a**) $\gamma_{\delta 2}$ exponent and (**b**) $\left(u_{\tau ,inl}/u_{\tau ,rec}\right)$ ratio via iLES.

**Figure 10:**
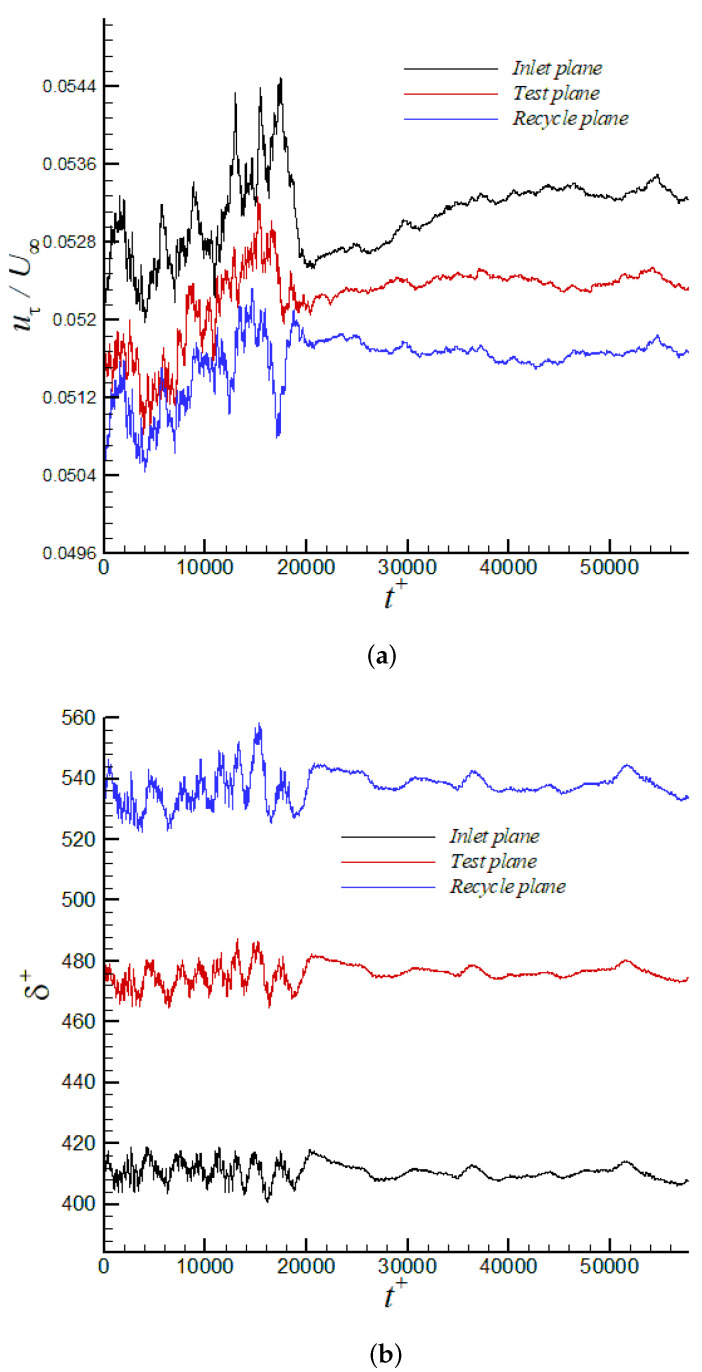
Time variation of (**a**) friction velocity normalized by freestream velocity and (**b**) boundary layer thickness in wall units at the inlet, test, and recycle plane via iLES.

**Figure 11:**
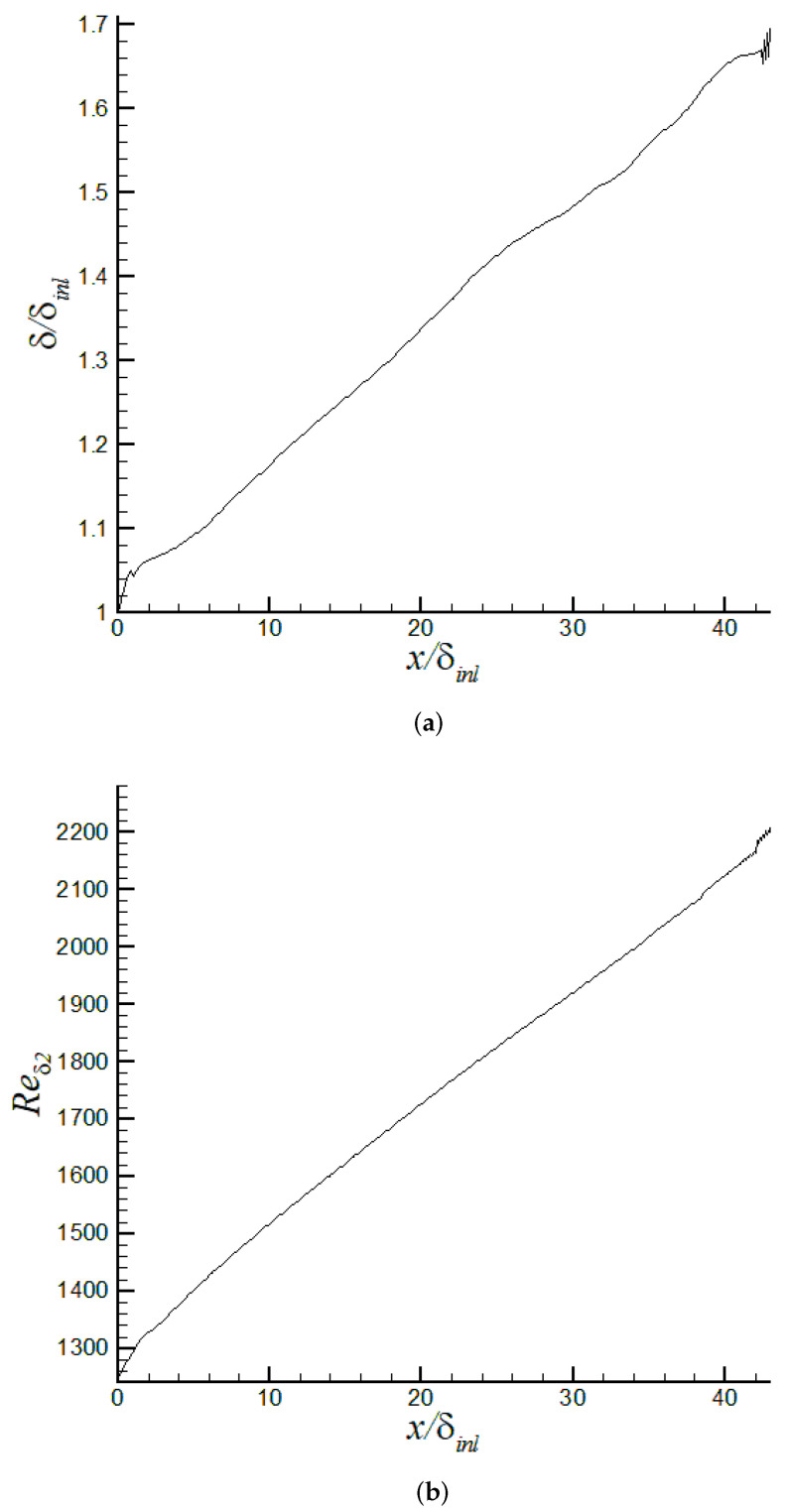
Streamwise development of (**a**) boundary layer thickness, $\delta /\delta_{inl}$, and (**b**) momentum thickness Reynolds number, $Re_{\delta 2}$, via iLES.

It is possible that the principal innovation of the present article is the implementation and evaluation of the turbulent inflow methodology based on DMA. Figure 9a shows the time variation of the power $\gamma_{\delta 2}$, as defined by Equation (9), for the iLES case. The power $\gamma_{\delta 2}$ oscillates significantly in the transient stage of the simulation. The case was run for approximately 64 flow-through time to ensure convergence of flow statistics, where the temporal filter for “on the fly” calculation of the mean flow was varied. As the flow reaches the “statistically steady state” (sample collection was performed during the last 30,000 viscous time, i.e., $t^{+}=t/\left(u_{\tau }^{2}/\nu_{w}\right)$, or last 35 flow-through time), the cumulative average value of the exponent is roughly −0.1081. This averaged power differs by around 13% from the typical empirical value of −1/8 as described by White [66]. On the other hand, the time-averaged $\gamma_{\delta 2}$ agrees quite well with regression (−0.105), as reported by [15], which is based on experiments by Coles, Mabey, and Shutts [68,69,70] at a Mach number range of $2.5<M_{\infty }<4.5$. The time-averaged ratio $\left(u_{\tau ,inl}/u_{\tau ,rec}\right)$ is slightly above one by the end of the predictions, as seen in Figure 9b. In Figure 10a, the temporal evolution of the near-wall region is explored via the friction velocity (proportional to the wall shear stress) at the inlet, test, and recycle stations. It can be observed that friction velocities are within 5.17% to 5.5% of the freestream velocity, $U_{\infty }$. The time variation of the edge of the boundary layer thickness in wall units is described by Figure 10b. Clearly, the proposed turbulent inflow generation [36,37,38] is demonstrated to be stable, being able to produce realistic inlet conditions. Similarly, the streamwise variation of the 99% boundary layer thickness, δ, and normalized by the inlet value is depicted in Figure 11a, where a very short developing section (∼2.5 to 3 $\delta_{inl}$’s) is observed. Downstream, the typical linear behavior of the boundary layer thickness in ZPG flows can be seen. By the end of the computational domain, some wiggles are present, caused by the prescribed zero-flux condition at the outflow. Furthermore, the momentum thickness Reynolds number, $Re_{\delta 2}$, in Figure 11b exhibits a very analogous streamwise trend to δ.

**Figure 12:**
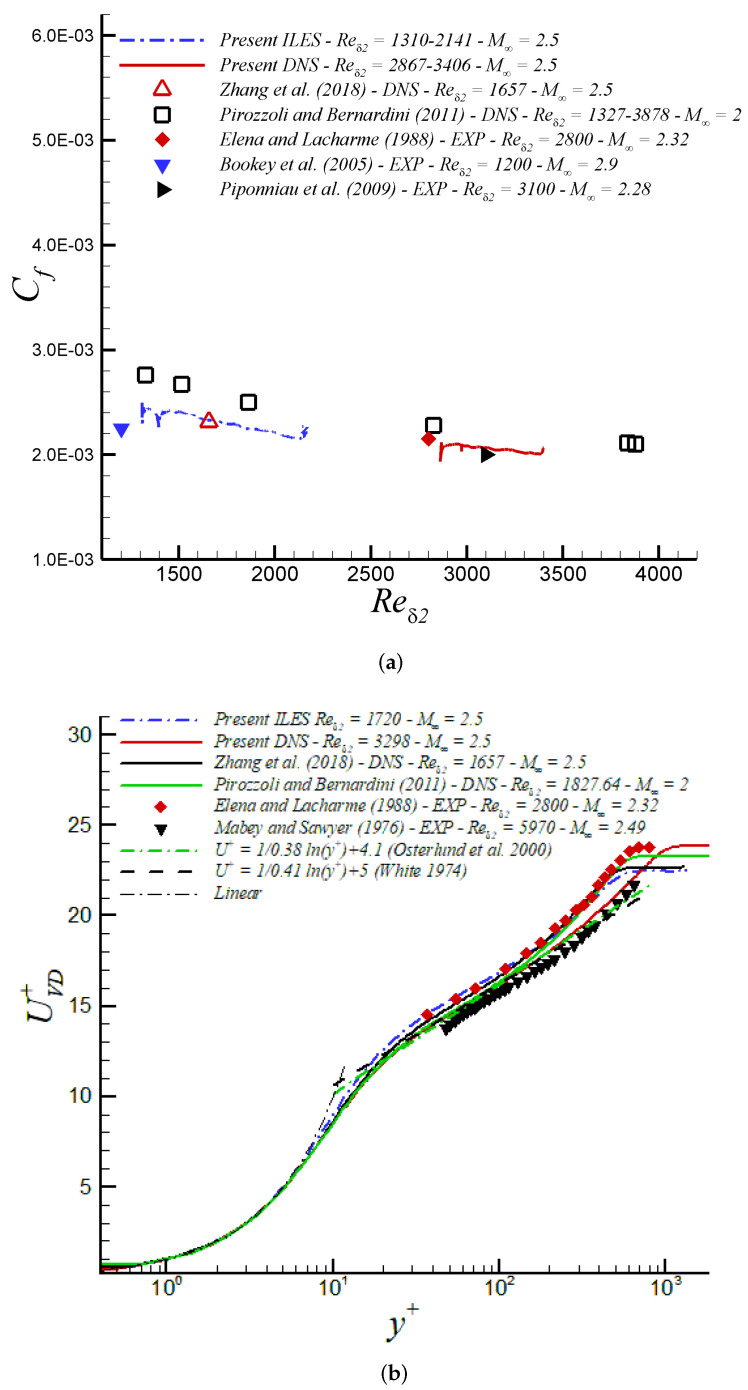
(**a**) Skin friction coefficient and (**b**) van Driest transformation in wall units at $M_{\infty }$ = 2.5 via iLES.

The skin friction coefficient, $C_{f}$, is depicted in Figure 12a vs. Reynolds numbers ($Re_{\delta 2}$). Our iLES approach exhibits excellent performance, showing good agreement with DNS from Zhang et al. [71]; however, we have prescribed 267 times fewer points (563 M vs. 2.1 M grid points) in our iLES domain with respect to the DNS domain in [71]. Note that the skin friction coefficient slope from iLES follows a similar tendency as our DNS’ slope [3] at higher Reynolds numbers. In both cases (i.e., in iLES and DNS), the corresponding turbulent inflow conditions based on the DMA approach have been very realistic and accurate according to the very short inlet developing section observed (≈$3\delta_{inlet}$${}^{\prime }s$ for reader’s reference). In [33], the inflow boundary condition was created by the compressible Blasius solution and turbulence transition was triggered via a body force method. While this approach in [33] may generate more natural inflow information, the total transitional developing zone encompassed approximately 30 reference boundary layer thicknesses, one order of magnitude larger than in the present study based on the DMA methodology. The mean streamwise velocity based on the van Driest transform from iLES exhibits excellent agreement with the DNS from Zhang et al. [71] at similar Reynolds and Mach number, almost overlapping, as well as with the DNS by [49] at $M_{\infty }$ = 2 and LDV measurements by [63] at $M_{\infty }$ = 2.32 (see Figure 12b). A clear Reynolds number dependency is present beyond $y^{+}>30$ in comparison with our DNS supersonic case at $Re_{\delta 2}$ = 3298 and experiments by Mabey and Sawyer (1976) at $Re_{\delta 2}$ = 5970. Furthermore, there is an obvious upward shift and shrinking of the logarithmic region given by the lower Reynolds numbers considered in iLES (also observed in DNS by [71]). However, the slope $\partial U_{VD}^{+}/\partial y^{+}$ exhibits a clear log behavior according to coefficients proposed by White [66] and Osterlund et al. [67] (≈100 wall units). The presence of a “hump” in the $U_{VD}^{+}$ profile for iLES around 20 $<y^{+}<$ 40 might be caused by a lack of numerical dissipation in this region, and further analysis should be carried out.

**Figure 13:**
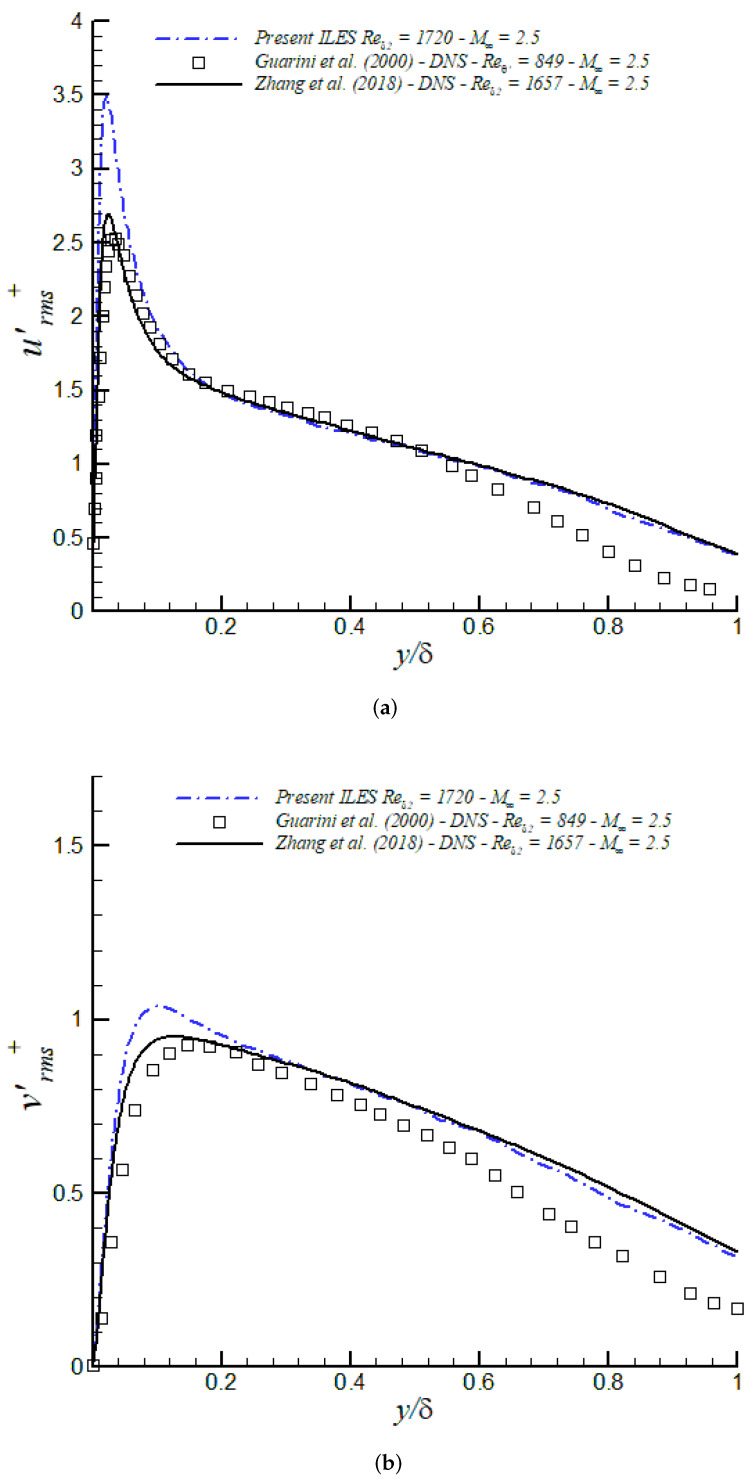

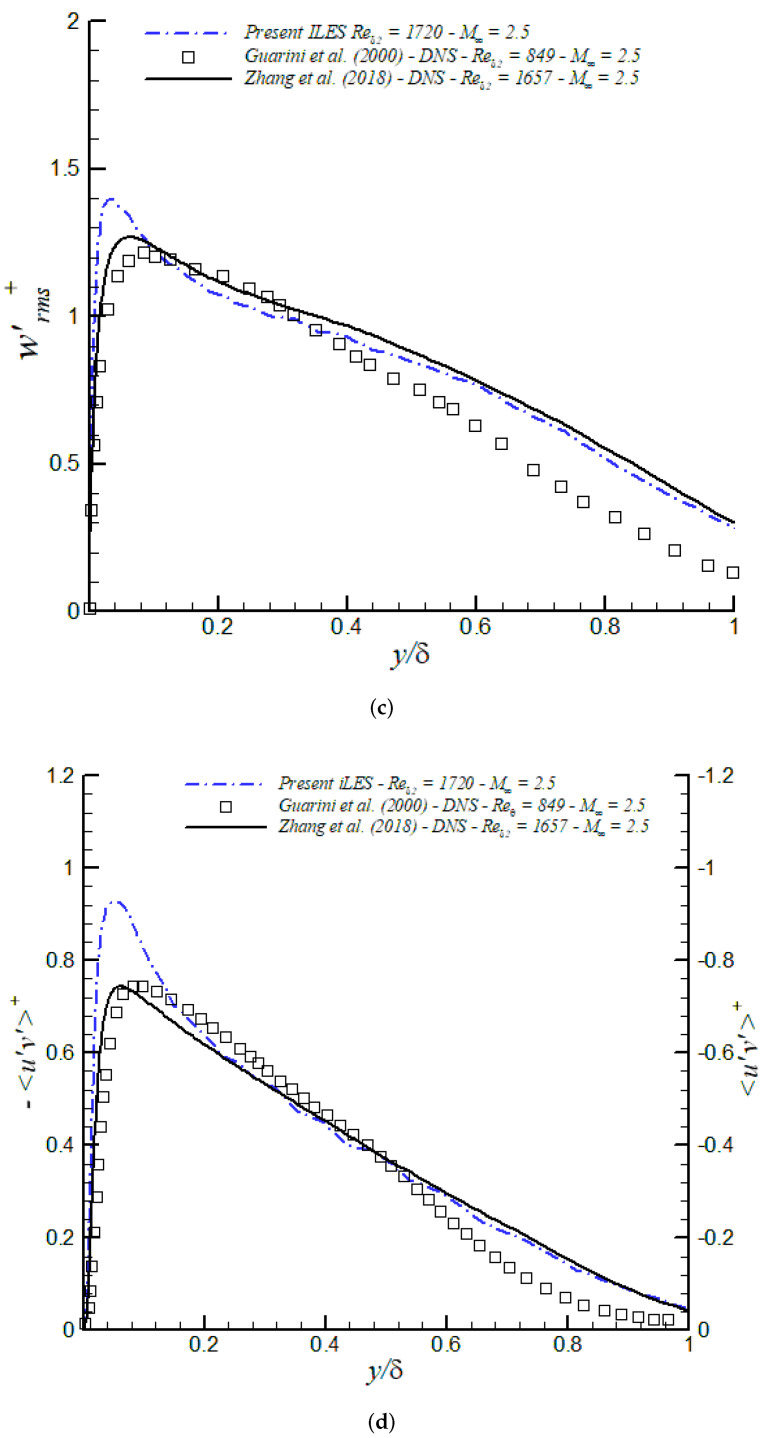
Turbulence intensities and Reynolds shear stresses in inner/outer units at $M_{\infty }$ = 2.5 via iLES: (**a**) $u_{rms}^{\prime }$, (**b**) $v_{rms}^{\prime }$, (**c**) $w_{rms}^{\prime }$, (**d**) $<u^{\prime }v^{\prime }>$.

Turbulence intensities ($u_{rms}^{\prime +}$, $v_{rms}^{\prime +}$, $w_{rms}^{\prime +}$) and Reynolds shear stresses ($<u^{\prime }v^{\prime }{>}^{+}$) are shown in Figure 13 together with DNS data at similar Reynolds numbers from [71,72]. Lessons learned are as follows:

(a) iLES significantly overpredicts peak values of u′, and the Reynolds shear stress peaks in the buffer layer.
(b) Excellent agreement of turbulence intensities and $<u^{\prime }v^{\prime }{>}^{+}$ in the outer region by iLES with respect to DNS from Zang et al. (2018) [71] at similar Reynolds numbers.

**Figure 14:**
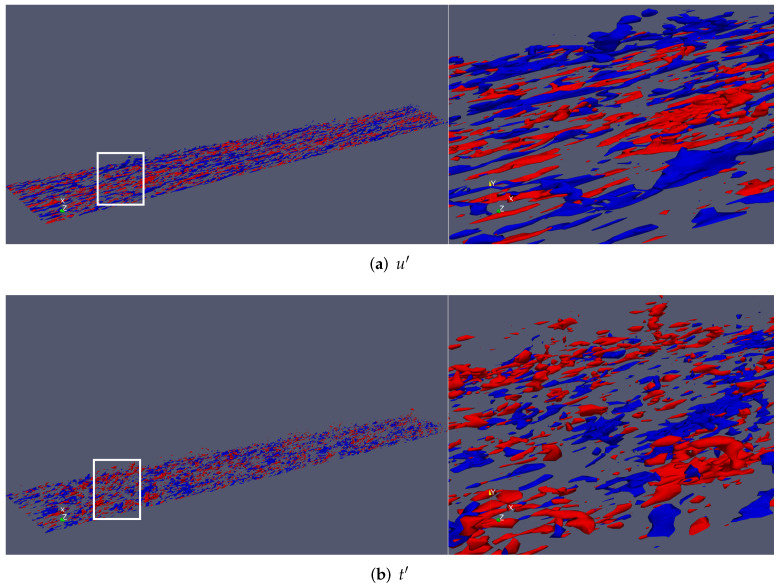
Iso-surfaces of streamwise velocity fluctuations (**a**) and thermal fluctuations (**b**) (positive values in red and negative values in blue).

Iso-surfaces of resolved streamwise velocity fluctuations ($u^{\prime }$) and thermal fluctuations ($t^{\prime }$) were extracted and are displayed in Figure 14. Positive values of fluctuations are in red, whereas negative values are represented by blue surfaces. Iso-surfaces of $u^{\prime }$ were extracted at 20% of the freestream velocity (±0.20$U_{\infty }$), while the wall temperature (maximum) was considered as a reference for thermal fluctuation extraction, i.e., $\pm 0.18T_{w}$. From Figure 14a, one can observe the presence of low (blue) and high (red) speed streaks, particularly in the near-wall region. A close-up window can be seen on the right. Low-speed streaks appear as very elongated parcels of fluid with low momentum. At such a moderate Reynolds number, the turbulence structure is finer and more isotropic with respect to lower Reynolds numbers (not shown here). Some strong ejection events ($u^{\prime }<0$, $v^{\prime }>0$) can be observed (or pumping up of low-momentum fluid by hairpin legs). On the other hand, sweep events ($u^{\prime }>0$, $v^{\prime }<0$) bring high-momentum fluid to the wall. Figure 14b exhibits iso-surfaces of static thermal fluctuations $t^{\prime }$. Overall, the thermal fluctuating field seems to be more isotropic, with a much finer structure of turbulence. The Reynolds analogy is somewhat satisfied but in the negative sense, i.e., $u^{\prime }t^{\prime }<$ 0, as reported by a number of investigators [35,49]. There are some regions of low-momentum fluid (or low-speed streaks), which are highly correlated to high (hot) thermal fluctuations and vice versa. Our experience dictates that the Reynolds analogy is much better satisfied in incompressible flow, indicating some compressibility effects at the supersonic regime.

## 4. Conclusions

Implicit LES (iLES) of supersonic ZPG spatially developing turbulent boundary layers has been carried out at moderate Reynolds numbers. The iLES accuracy has been demonstrated by contrasting the present results against theory, DNS, and experimental data. A weak compressibility effect on turbulent thermal structures based on two-point correlations has been found. The Reynolds analogy ($u^{\prime }$ vs. $t^{\prime }$) approximately holds for the supersonic regime, but to a lesser extent, as observed in incompressible turbulent boundary layers with temperature as a passive scalar. A more prolonged power law behavior of the mean streamwise velocity in the outer region was found compared to the log law at Mach 2.5. Implicit LES has shown excellent performance in Mach 2.5 adiabatic flat plates in terms of the mean flow (i.e., $C_{f}$ and $U_{VD}^{+}$).

iLES significantly overpredicts peak values of u′, and consequently, Reynolds shear stress peaks in the buffer layer. Excellent agreement of turbulence intensities and Reynolds shear stresses was achieved in the outer region by iLES regarding external DNS databases at similar Reynolds numbers. The total computational resource saving was estimated to be roughly in the order of 100 for our DNS database and in the order of 1200 with respect to DNS by [71]. An ongoing examination of the standard dynamic Smagorinsky SGS model is being conducted.

## Data Availability

Not applicable.
